# The accuracy of femoral component rotational measurements using computed tomography—a cadaveric study

**DOI:** 10.1186/s42836-020-00052-6

**Published:** 2020-12-03

**Authors:** Onyedikachi Eseonu, Calum Cree, Martin Sambrook, Mark Blyth, Bryn Jones

**Affiliations:** 1 Department of Trauma and Orthopaedic Surgery, Glasgow Royal Infirmary, Castle Street, Glasgow, G4 0SF UK; 2 Department of Trauma and Orthopaedic Surgery, Dumfries and Galloway Royal Infirmary, Cargenbridge, Dumfries, DG2 8RX UK; 3 Department of Radiology, Eastbourne District General Hospital, Kings Drive, Eastbourne, BN21 2UD UK

**Keywords:** Total knee arthroplasty, Total knee replacement, Rotation, Computed tomography, Cadaver

## Abstract

**Background:**

CT scans can be used to assess the rotational alignment of the femoral component following total knee arthroplasty (TKA). This is done by calculating the posterior condylar angle (PCA).

However, the methods used may not account for the biomechanical functionality of the TKA components. This cadaveric study aimed to determine whether the axis of scanning (mechanical or anatomical) alters the results of PCA calculations.

**Methods:**

CT scans of 12 cadaveric adult femora were performed along the anatomical axis and the mechanical axis. The PCA was determined on each CT scan by measuring the relationship of the prosthetic posterior condyles to the surgical epicondylar axis of the femur. The mechanical and anatomical axis groups were further subdivided into best-fit and multi-slice subgroups. As a control, the posterior condylar angle was also calculated on photographic images of each femur. Bland-Altman plots were used to determine the correlation between the PCA values obtained from the different scanning axes and measurement techniques.

**Results:**

There was no significant difference between the PCA measurements derived from anatomical and mechanical axis CT scans. The Pearson correlation co-efficient also indicated good correlation between the two scanning axes.

**Conclusion:**

The axis of scanning does not significantly affect the PCA measurements. Therefore, the measurements may be reliably used for clinical decision-making, regardless of the axis of CT scanning.

## Background

Total knee arthroplasty (TKA) has a patient satisfaction rate of approximately 80% [1–3]. Accurate rotational alignment of the components is an important prognostic factor in TKA. Component malrotation can cause patellar maltracking and altered tibiofemoral kinematics, which contributes to post-operative pain and abnormal loading [4–14], predisposing to early implant failure and revision surgery.

In patients presenting with knee pain, knee instability or early loosening post TKA, CT scans can be used to assess the rotational alignment of the components. Using the Berger protocol, femoral component rotation can be determined by the prosthetic posterior condylar angle (PCA) [6]. The PCA is the angle formed by two lines drawn on an axial CT image. The first is the surgical epicondylar axis (SEA) between the lateral epicondyle and the medial sulcus (immediately posterior to the medial epicondyle). The second is the prosthetic posterior condylar line (PCL) between the surfaces of the medial and lateral posterior condyles of the prosthesis.

Berger *et al* [6] measured the PCA on axial CT images taken perpendicular to the anatomical axis of the femur (anatomical axis scanning). However, axial CT scans of the femur, performed perpendicular to the axis of function of the femur (mechanical axis scanning), may be more biomechanically relevant in a painful TKA. Scanning along different axes may alter the perceived relationship between the anatomical landmarks for the SEA and PCL, which may alter the PCA value derived from these lines. Berger *et al* [6] also measured the PCA on a single CT slice that best showed both epicondyles (best-fit technique). However, with advancements in radiological software, it is now possible to mark an anatomical landmark on the slice that it is best displayed and superimpose this marker onto a different CT slice where another relevant landmarked is best displayed (multi-slice technique), arguably allowing for more accurate measurements. Since the PCA contributes to decision making on revision surgery in symptomatic TKAs, it is important for clinicians to have biomechanically valid and accurate PCA measurements. Until now, no studies have investigated whether there is any difference between PCA measurements derived from anatomical axis scanning *vs*. mechanical axis scanning, and those derived by the original best-fit technique *vs*. the newer multi-slice technique.

This study aimed to determine if there was any difference between the PCA measurements derived from images of the same femur when scanning along both the mechanical and anatomical axes. In addition, this study sought to compare the PCA derived from measurements via the best fit and multi-slice techniques and measure any differences between these techniques.

## Methods and materials

### Materials

Twelve cadaveric femora (5 right, 7 left) were obtained from donors who bequeathed their body to the Anatomy Facility at the University of Glasgow for educational and research purposes. A specialist knee surgeon passed a hypodermic needle through a radio-opaque marker into the medial sulcus and lateral epicondyle of the femora to allow consistent photographic and radiological identification of these landmarks. Each femur was numbered to prevent duplication.

### Calculation of photographic PCA

**Fig. 1:**
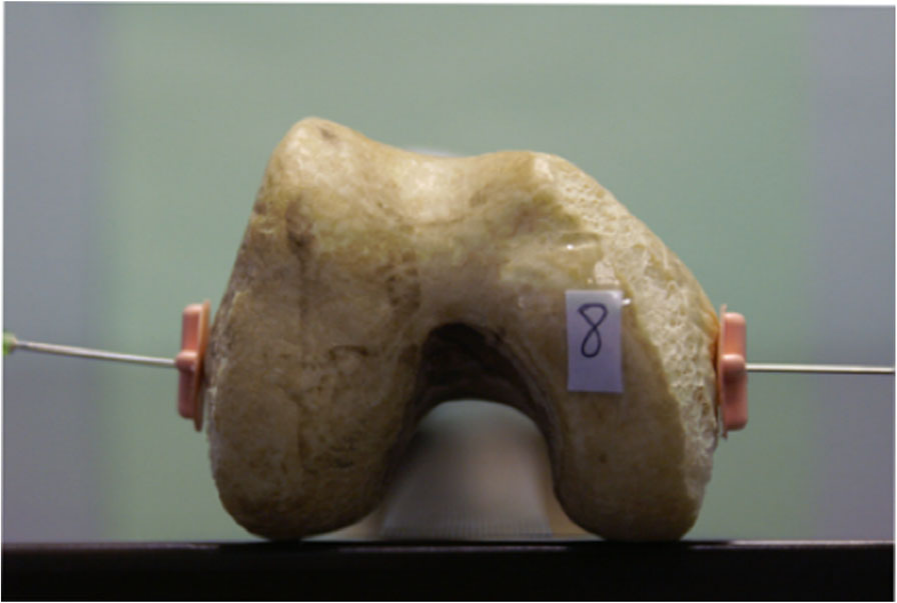
Photographic image along femoral mechanical axis

**Fig. 2:**
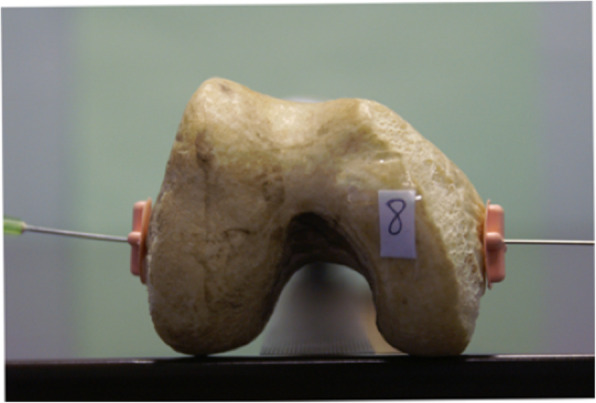
Photographic image along femoral anatomical axis

The PCA was calculated on photographic images of each femur as a control group. A simulated axial view of each femur was achieved by digitally photographing the distal aspect of all 12 femora along the mechanical and anatomical axes (Figs. 1 and 2). These photographic images were imported to Adobe Photoshop 8.0 (Adobe Systems Incorporated, San Jose, CA). The SEA and PCL were identified and marked on the photographic images of each femur by a single observer according to the method described by Berger *et al* [6]. The angle subtended by the SEA and PCL was measured to give the PCA for each femur.

### CT calculation of PCA

Using a Siemens Somatom Sensation 4 CT scanner (Siemens, Munich, Germany), we scanned each femur twice, along the anatomical axis and mechanical axis, to obtain 1.5-mm thick slices. The radiological markers of the medial sulcus and lateral epicondyle were left on during scanning to allow reproducible identification of these points.

The CT images were evaluated on Philips digital imaging and communications in medicine (DICOM) software by using the best-fit technique (measurments made on a single CT slice that best showed both epicondyles). In order to minimize intra-oberver error, the measurements were repeated 10 times for each femur to produce a mean PCA.

### PCA on best fit vs multi-slice CT

The mechanical and anatomical axis groups were further subdivided into best-fit and multi-slice subgroups. In the best-fit subgroup, the best-fit method was followed 10 times, for both the anatomical and mechanical axis scans of each femur to produce a mean PCA for each group. In the multi-slice subgroup, the lateral epicondyle and medial sulcus were marked on the CT slices that they were best displayed. These markings were superimposed onto all the other CT slices. The SEA was drawn between the two marks, allowing the PCA to be calculated. The PCAs obtained for the best-fit and multi-slice methods were then directly compared for each scanning axis.

### Statistical analysis

Bland-Altman plots were used to compare the PCAs obtained from the different scanning methods and the best fit/multi-slice subgroups. The mean of the PCA measurements from both measurement methods (X-axis) were plotted against the difference between the two measurement techniques (Y-axis). When the difference between the two methods did not exceed the 95% confidence interval (CI) limits of agreement (mean difference ±2 standard deviations) when plotted on the Bland-Altman plot, the two measurement methods were considered to be in agreement and interchangeable. Pearson correlation coefficients (PCC) were also calculated using the Statistical Package for the Social Sciences (SPSS) version 24.0 for Windows (IBM Corp., Armonk, New York) to determine the degree of correlation between the PCA values obtained by the different measurement methods.

## Results

### Comparison of photographic control and CT PCA measurements

#### Mechanical Axis

**Fig. 3:**
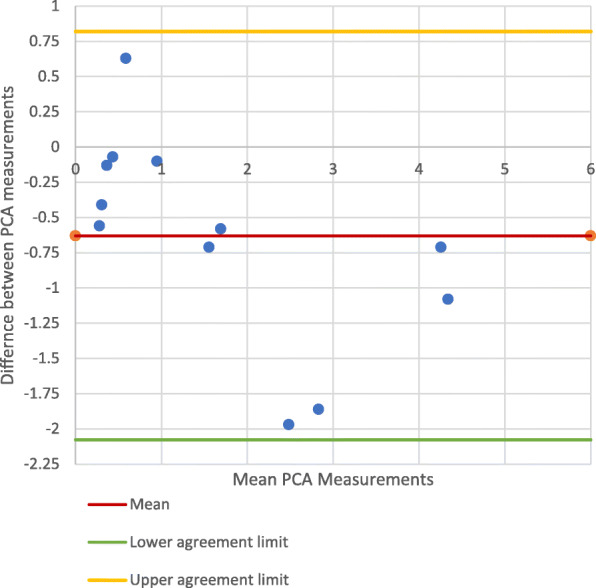
PCA measurements on mechanical axis-photographic image *vs*. CT best-fit technique

**Fig. 4:**
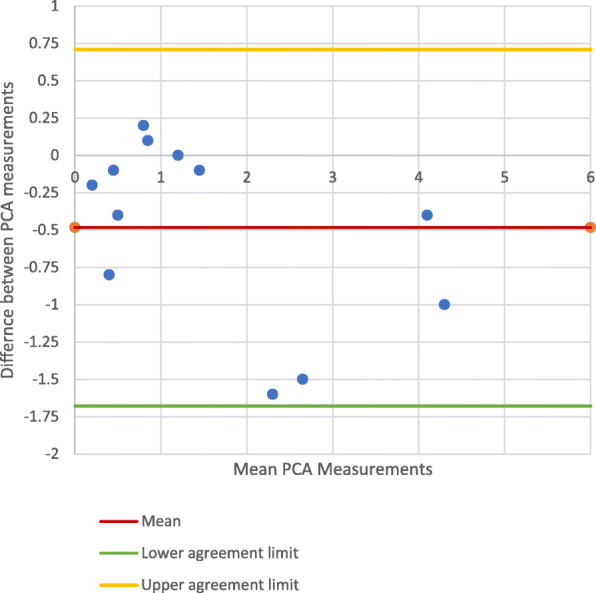
PCA measurements on mechanical axis-photography *vs*. CT multi-slice technique

Figures 3 and 4 show the Bland-Altman plots comparing the PCA measurements from the mechanical axis control photographic images and mechanical axis CT images. The points on these plots all lie within the 95% CI (Best-fit: − 2.078 - 0.8200; Multi-slice: − 1.677 - 0.710), indicating there was no significant difference between the PCA measurements made on the CT images and control photographs. The PCCs (Best-fit: 0.923; Multi-slice: 0.933) demonstrated high correlation between the control photographic measurements and the CT measurements.

#### Anatomical Axis

**Fig. 5:**
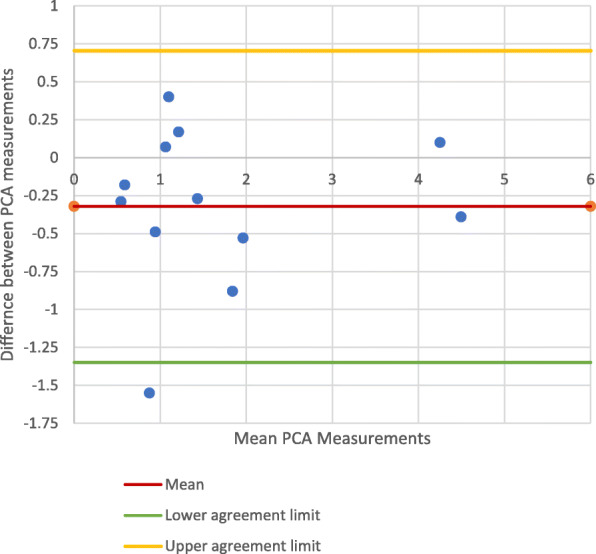
PCA measurements on anatomical axis-photography *vs*. CT Best fit technique

**Fig. 6:**
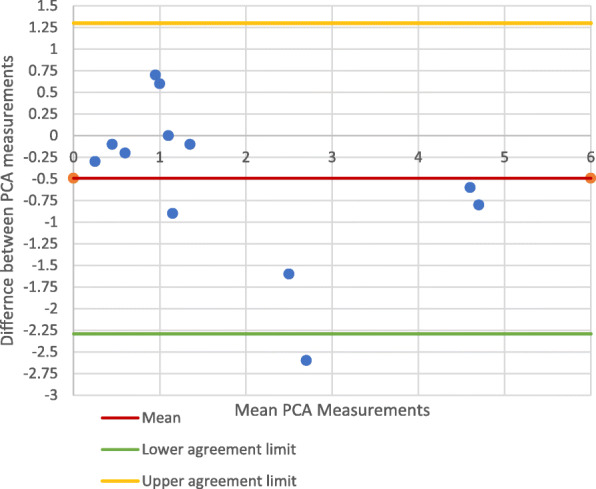
PCA Measurements on anatomical axis-photography *vs*. CT multi-slice technique

The points on the Bland-Altman plots comparing the PCA measurements from the anatomical axis control photographs and anatomical axis CT images did not all lie within the 95% CI (Best fit: − 1.346 - 0.706; Multislice: − 2.287 - 1.304), for both the best-fit (Fig. 5) and multi-slice techniques (Fig. 6). Therefore, although the PCCs (Best fit: 0.925; Multislice: 0.864) demonstrated good correlation between the photographic control and CT measurements, the plots showed that there was a significant difference between them.

### Anatomical *vs*. mechanical axis PCA measurements on CT images

**Fig. 7:**
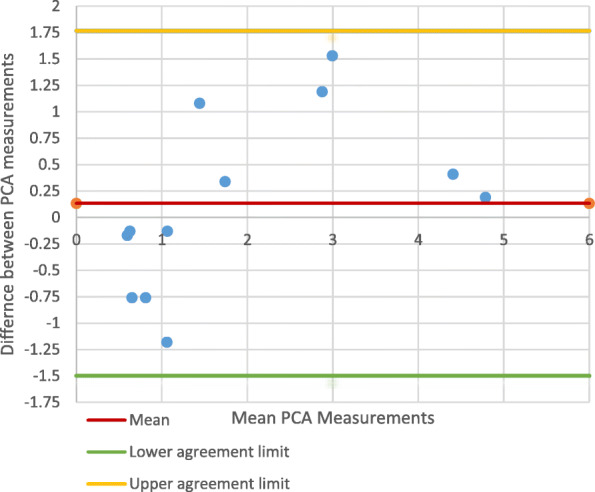
PCA Measurements on CT: anatomical *vs*. mechanical axis (Best fit technique)

The Bland-Altmann plot comparing the PCA measurements from anatomical *vs*. mechanical axis CT images (Fig. 7) showed that there was no significant difference between the scanning axes (95% CI: − 1.499-1.767). The PCC of 0.888 indicated good correlation between the two groups.

### Best-fit *vs*. multi-slice PCA measurements on CT images

**Fig. 8:**
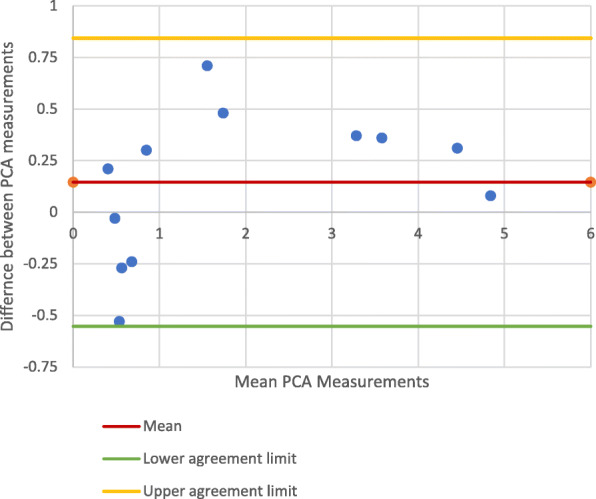
PCA Measurements on Mechanical axis CT scanning - Best fit vs Multl-slice technique

**Fig. 9:**
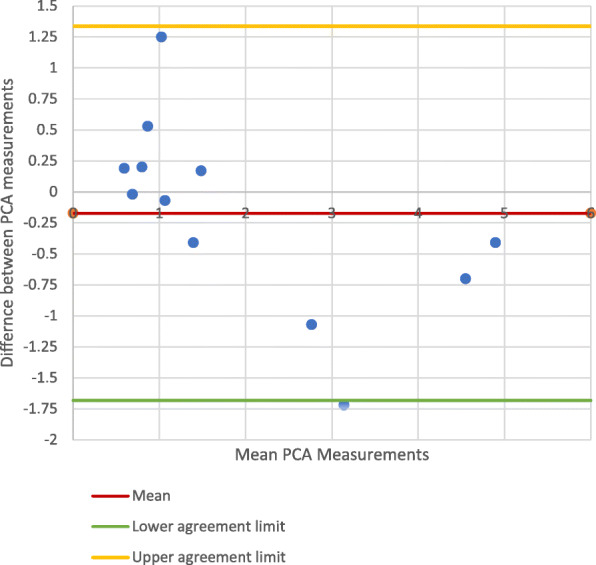
PCA Measurements on anatomical axis CT scanning: best-fit *vs*. multl-slice method

Comparison of the PCA measurements showed good correlation (PCC: 0.981) and no significant difference between the best fit and multi-slice techniques (Fig. 8) when scanning along the mechanical axis (95% CI: − 0.552 -0.844). When scanning along the anatomical axis however, there was a significant difference between the best-fit and multi-slice techniques (Fig. 9) (95% CI: − 1.682-1.338). The PCC of 0.920 suggests that there was still good correlation between the measurement methods.

## Discussion

This study investigated two important issues related to the measurement of TKA component rotation, namely, (1) the femoral scanning axis and (2) the traditional best-fit *vs*. multi-slice technique, and how the two methods might influence the accuracy of painful TKA assessment.

The photographic controls represented the gold standard for landmark identification, as the radiological marker pins were directly placed on the relevant anatomical landmarks. Our results demonstrated that mechanical axis CT scanning more accurately replicated the gold standard than the anatomical axis scans. However, there was no statistically significant difference in the PCA measurements when directly comparing the anatomical and mechanical axes, suggesting that the axis of scanning may not have an effect on PCA measurements.

It is unclear, from our results, whether the newer multi-slice technique for PCA measurement is significantly superior to the original best-fit method, because there was no significant difference between the techniques on the mechanical axis scanning. However, a significant difference was identified on anatomical axis CT images.

The lack of a significant difference between anatomical *vs*. mechanical axis scanning, and the best-fit *vs*. multi-slice technique implies that there is no need for specific positioning protocols when obtaining CT scans for assessment of a painful TKA. It also implies that PCA measurements derived without the use of advanced radiological software (e.g. in resource poor environments) can be relied upon for patient management.

Our study has shed light on some important issues related to PCA measurement. However it also had some limitations. The study had a good number of femora for a cadaveric study. Nonetheless, a larger study, involving more femora may have been able to identify any possible differences between these techniques that out sample could not identify. In addition, all CT measurements were performed by a single observer. Although this single observer was experienced in interpreting CT scans, measurements by multiple observers would have improved the reliability of our results.

## Conclusion

The axis of CT scanning may not affect the PCA measurements derived, so PCA measurements can be used in clinical decision-making, regardless of the axis of scanning.

## Data Availability

Data used and/or analysed during the current study are available from the corresponding author on reasonable request.

## Abbreviations

TKA: Total knee arthroplasty
CT: Computed tomography
PCA: Posterior condylar angle
SEA: Surgical epicondylar axis
PCL: Prosthetic posterior condylar line
DICOM: Digital imaging and communications in medicine
CI: Confidence interval
PCC: Pearson Correlation Coefficients
SPSS: Statistical package for the Social Sciences
